# Competitive Adsorptive Mechanism of H_2_/N_2_ in LTA/FAU Zeolites by Molecular Simulations and Experiments

**DOI:** 10.3390/molecules29153686

**Published:** 2024-08-03

**Authors:** Zixu Dong, Zhilu Wang, Lina Zhang, Qiang Fu, Ming Wang

**Affiliations:** School of Chemistry and Chemical Engineering, Shandong University of Technology, Zibo 255049, China; 21406020666@stumail.sdut.edu.cn (Z.D.); sevenwangzl@163.com (Z.W.); zln20000521@163.com (L.Z.)

**Keywords:** adsorption mechanism, H_2_/N_2_ adsorptive separation, adsorption site

## Abstract

For industrial tail gas to be converted into high-purity hydrogen, the H_2_-N_2_ mixture needs to be separated efficiently. This work examined the adsorption characteristics and competitive mechanisms of H_2_ and N_2_ on LTA- and FAU-type zeolites, at 77 K, 298 K, and 0.1–10 bar by thoroughly analyzing results of adsorption capacity experiments and molecular simulations. In the Grand Canonical Monte Carlo (GCMC) simulations, the force field causing a molecular dipole of H_2_ and the polarization force field of N_2_ are first applied. The accuracy of the force field was experimentally verified. The findings indicate that N_2_ and H_2_ loading on Ca-FAU (Ca-LTA) are higher than Na-FAU (Na-LTA). On NaX at 77 K, the highest adsorption selectivity (N_2_/H_2_) is observed; on NaA at 298 K, it is the opposite. The GCMC data findings demonstrate that H_2_ and N_2_ have remarkably similar adsorption sites, with framework oxygen atoms and non-framework cations serving as the main adsorption sites for adsorbate molecules. Furthermore, the rate at which H_2_ diffuses is higher than that of N_2_. The study of redistribution charge before and after adsorption demonstrated that N_2_ has a greater affinity for the framework oxygen atoms than H_2_. This study provides a molecular theoretical foundation for the adsorption behavior of H_2_-N_2_ mixture in zeolites.

## 1. Introduction

H_2_ is a green and clean energy source with advantages of cleanliness, no pollution, zero emissions, and wide applications. The heat of the combustion of hydrogen is higher than that of most fuels based on the same mass, making it an ideal substitute for fossil fuels. According to sources, hydrogen can be divided into gray hydrogen [1,2,3], blue hydrogen [4,5], green hydrogen [6], and hydrogen-rich tail gas [7,8]. Currently, over 95% of hydrogen generation occurs through steam methane reforming (SMR), resulting in what is commonly referred to as “gray hydrogen”, or via coal gasification, which produces “brown hydrogen”. A promising approach toward decarbonized hydrogen involves combining SMR or gasification with carbon capture and sequestration (CCS), resulting in “blue hydrogen”. If renewable or nuclear power is used to supply the electricity for this process, the resulting hydrogen would be decarbonized and recognized as “green hydrogen” [9]. While gray hydrogen production currently accounts for a high proportion, it does result in significant pollution due to the discharge of harmful gasses. Furthermore, although environmentally friendly, green hydrogen can be costly, and its application rate is currently limited. However, the recovery of hydrogen-rich tail gas offers significant benefits for the comprehensive utilization of hydrogen and carbon neutrality. Currently, there is significant interest in the industrial exhaust effect caused by processes such as steam methane reforming (H_2_/N_2_/CO_2_) [10,11], ammonia production (H_2_/N_2_/NH_3_) [12], and coke oven gas (H_2_/N_2_/CH_4_) [13]. These processes result in the production of a substantial amount of H_2_-N_2_ mixture. As a result, many researchers have focused on binary or ternary mixtures containing richer impurities such as CO_2_/CH_4_ [14]. According to Carbajo’s study on the adsorption and separation behavior of gas mixtures such as H_2_/N_2_/CO_2_/CH_4_/CO in zeolites, H_2_ or N_2_ had a lesser interaction with zeolite framework atoms [15]. This made it harder to separate them from other gasses compared to those gasses with higher polarizability. The associated physical properties of N_2_ and H_2_ gasses are shown in Table 1.

Yaremov et al. investigated the effects of microporous zeolites’ pore parameters and surface characteristics (at 77, 197, and 253 K; 760 torr) on the adsorption of H_2_, N_2_, CO, and CH_4_. According to this study, the adsorption inertia of H_2_ helped in gas separation from other gasses [16]. There is research on the separation of H_2_-N_2_ mixture predominantly using MOFs [17], activated carbons [18,19,20], membranes [21,22], and zeolites [23]. A zeolite is a kind of porous material widely used in industry. It can selectively adsorb molecules based on their size, shape, and polarity. Li et al. studied N_2_ adsorption in Linde Type A (LTA) and truncated octahedron (FAU)-type zeolites at 1 bar and 300 K [24,25]. The results indicated that N_2_ had superior adsorption performance in FAU-type zeolites, with an adsorption capacity of up to 16.26 mol/kg. Then, the N_2_ adsorption capacity of the LiX zeolite was twice that of the Type A zeolite with extra-framework cations of Ca (CaA) zeolite [26]. The results highlighted the benefits of FAU- and LTA-type zeolites for adsorptive H_2_ or N_2_. However, the study of the competitive adsorption mechanism of H_2_-N_2_ mixture in FAU- and LTA-type zeolites is not clear. Most researchers have utilized molecular simulation techniques employing classical force fields to investigate the adsorption of H_2_ or N_2_ in zeolites. In general, simulations employing classical force fields fail to replicate experimental findings. To address the inaccuracy of classical force fields, we examined the accuracy of hydrogen force field parameters, specifically dipole moments, and incorporated polarization correction in the nitrogen force field parameters. The simulation results for single-component adsorption isotherms exhibit strong agreement with experimental test results, confirming the credibility of the force field parameters and the models. This paper analyzes the effects of cation type (Na^+^ and Ca^2+^), adsorption temperature (77 K and 298 K), and pressure (0–10 bar) on the adsorption capacity of H_2_ and N_2_ in LTA- and FAU-type zeolites using the force field parameters. In addition, it examines the competitive diffusion behavior of adsorbate molecules and investigates the law of competitive diffusion. Furthermore, the competitive adsorption mechanism of H_2_ and N_2_ in zeolites is examined in terms of the adsorption sites and the charge density.

## 2. Results and Discussion

### 2.1. Adsorption Isotherm

**Figure 1:**
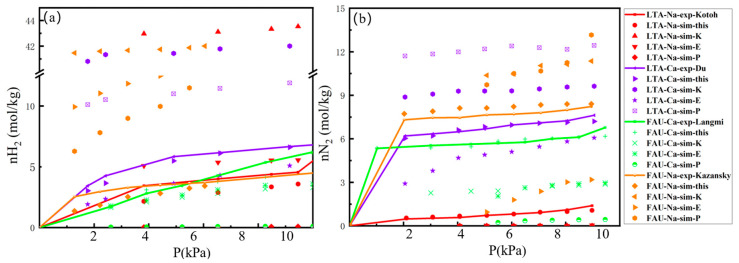
Adsorption isotherms of H_2_ (**a**) and N_2_ (**b**) in NaA, CaA, NaX, and CaX zeolites at 77 K, 0–1 bar, and the reported force fields by K^44^, E^43^, P^46^, G3^42^, V^41^, and H^47^.

**Figure 2:**
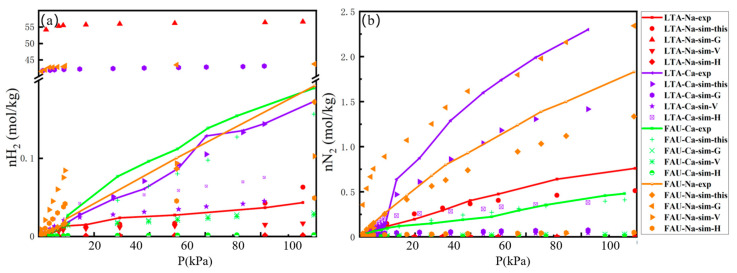
Adsorption isotherms of H_2_ (**a**) and N_2_ (**b**) in NaA, CaA, NaX, and CaX zeolites at 298 K, 0–10 bar, and the reported force fields by K^44^, E^43^, P^46^, G3^42^, V^41^, and H^47^.

The adsorption isotherms of H_2_ and N_2_ on LTA- and FAU-type zeolites were measured at 77 K (0.1–1 bar) and 298 K (0.1–10 bar). We conducted experimental H_2_ and N_2_ adsorption isotherms in the zeolite at 298 K, and the H_2_ isotherms at 77 K were obtained from the works of Kotoh [27], Du [28], Langmi [29], and Kazansky [30], while the N_2_ isotherm at 77 K was obtained from Sircar [31]. First, the one-component computed isotherms of H_2_ and N_2_ were compared with the available experimental data. It was observed that the simulated isotherms using the previously developed force field do not fit the experimental data shown in Figure 1 and Figure 2, and Evagelia used Feynman–Hibbs to explain the H_2_ quantum effect involving dispersion-type interactions in their force field [32]. However, all the adsorption isotherms from this study’s force field, as shown in Figure 1 and Figure 2, exhibit reasonable agreement between experiments and simulations, and using experience, the values of relative average deviation (RAD) lower than 30% are acceptable, as shown in Table S1. The simulated isotherms of H_2_ and N_2_ at 77 K slightly over-predict the adsorption isotherms for NaX. Nevertheless, the simulated isotherms of H_2_ and N_2_ at 298 K slightly under-predict the adsorption isotherms below 60 kPa for NaA. By fitting the adsorption isotherms results with Langmuir model, the Langmuir model parameters for H_2_ and N_2_ were estimated, and they are presented in Table S2. The adsorption isotherms depicted in Figure 1 and Figure 2 conform to the Langmuir isotherm, indicating that the adsorption of H_2_ and N_2_ on the LTA- and FAU-type zeolites follows a microporous adsorption behavior. In the 0–1 bar range at 77 K, the N_2_ loading on the LTA- and FAU-type zeolites is approximately twice the value of H_2_ loading, as shown in Figure 1a,b. However, in the 0–10 bar range at 298 K, the N_2_ loading on the LTA- and FAU-type zeolites exceeds ten times the H_2_ loading values shown in Figure 2a,b. It is observed that the adsorption value of H_2_ is greater in the FAU zeolite than in the LTA-type zeolite at 298 K. Furthermore, for FAU- and LTA-type zeolites at 77 K, the H_2_ and N_2_ adsorption capacities of Ca-FAU (Ca-LTA) are greater than those in Na-FAU (Na-LTA). In most cases, the adsorption affinity of H_2_ in Ca-FAU (Ca-LTA) is stronger than that of Na-FAU (Na-LTA), which can be attributed to the smaller radius of Na^+^ and the shorter interaction distance with adsorbent molecules. When Na^+^ is exchanged with Ca^2+^ in the zeolite, the non-framework cation decreases, therefore expanding the pore volume of the zeolite framework and allowing for an increase in the space available for H_2_ adsorption.

### 2.2. Adsorption Selectivity and Heat

Adsorption selectivity is a vital indicator used to evaluate the adsorption performance of adsorbent materials. In order to explore the H_2_ and N_2_ adsorption properties in LTA- and FAU-type zeolites, we apply the ideal adsorption solution theory (IAST) to predict the selectivity of H_2_-N_2_ mixture. The selectivity is calculated using Equation (1):

(1) $$S=\frac{q_{1}/q_{2}}{p_{1}/p_{2}}$$

where *q*_1_ and *q*_2_ are the adsorption capacities, and *p*_1_ and *p*_2_ are the partial pressures.

**Figure 3:**
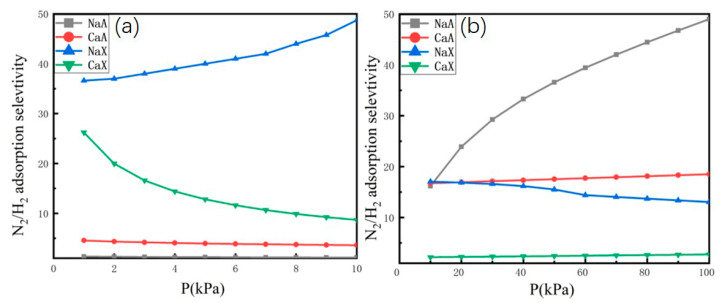
The adsorption selectivity for N_2_ over H_2_ in NaA, CaA, NaX, and CaX, which corresponds to the binary H_2_-N_2_ mixture (H_2_/N_2_, 50/50, *v*/*v*): (**a**) 77 K, (**b**) 298 K.

In this study, the selectivity of N_2_/H_2_ was predicted at 77 K within the range of 0–1 bar, and at 298 K within the range of 0–10 bar. Using the same molar fractions of H_2_ and N_2_, we can easily investigate the competitive adsorption behavior of the H_2_-N_2_ mixture. All calculations were performed using the PyIAST package (3.12.4) [33]. As shown in Figure 3, the selectivity of N_2_/H_2_ in the FAU-type zeolite is higher than that in the LTA-type zeolite at 77 K. Additionally, the selectivity of N_2_/H_2_ in Ca-FAU (Ca-LTA) is lower than that in Na-FAU (Na-LTA) at 298 K. The greatest selectivity of N_2_/H_2_ is observed in NaX at 77 K, while the greatest selectivity of N_2_/H_2_ is observed in NaA at 298 K. The adsorption selectivity from the binary N_2_-H_2_ mixture via GCMC simulations is shown in Figure S1. The results indicate that the selectivity values obtained from binary mixture GCMC simulation are consistent with those predicted using the IAST.

**Figure 4:**
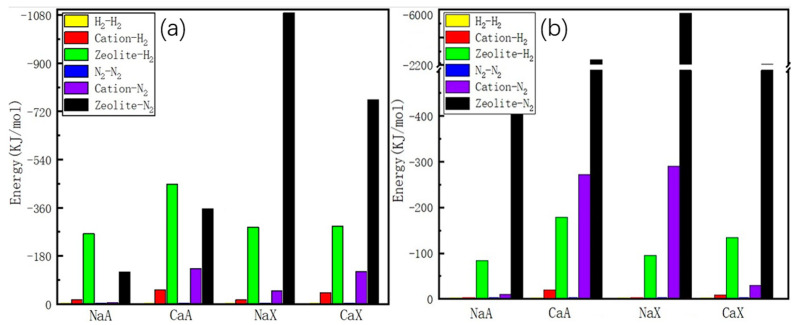
Adsorption energy of H_2_ and N_2_ in NaA, CaA, NaX, and CaX at 1 bar: (**a**) 77 K, (**b**) 298 K.

In Figure 4, we present contributions from different interactions to the energy of adsorption of H_2_ and N_2_ in FAU- and LTA-type zeolites at high loadings though MC calculations. It is evident that the primary contribution to the energy of adsorption for H_2_ and N_2_ is the significant host–guest interaction, which consists of a dispersive and an electrostatic component. The main difference is that N_2_ exhibits higher electrostatic interaction energies compared to H_2_, while they have similar dispersive contributions. In the simulations of the FAU- and LTA-type zeolites, the calculated energy of adsorption is primarily influenced by the adsorption site binding energy, with only a minor contribution from the guest–guest interaction. However, the guest–guest interaction is approximately zero since we used a one-site guest model. To investigate the effect of temperature on adsorption, we conducted the calculation using single-component gas equilibrium adsorption isotherms at 77 K and 298 K, according to the Virial Method [34]. The heat of adsorption at infinite dilution is provided in Table 2.

### 2.3. Adsorption Sites

**Figure 5:**
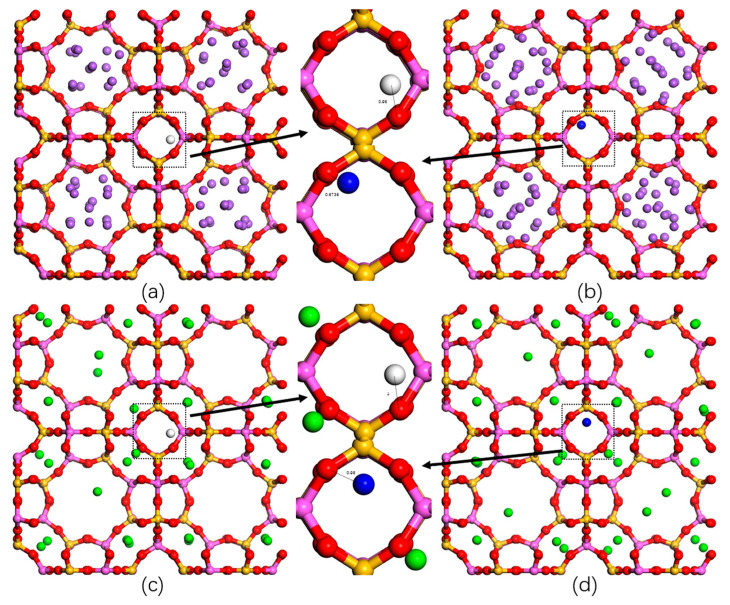
Typical binding geometry of H_2_ and N_2_ adsorbed in the super cage of the zeolite employed according to our computer simulations at 77 K. (**a**,**b**) NaA, (**c**,**d**) CaA, H_2_ molecule (white), N_2_ molecule (blue), Si-zeolite (pink), Al-zeolite (yellow), O-zeolite (red), Na^+^ cation (purple), and Ca^2+^ cation (green).

**Figure 6:**
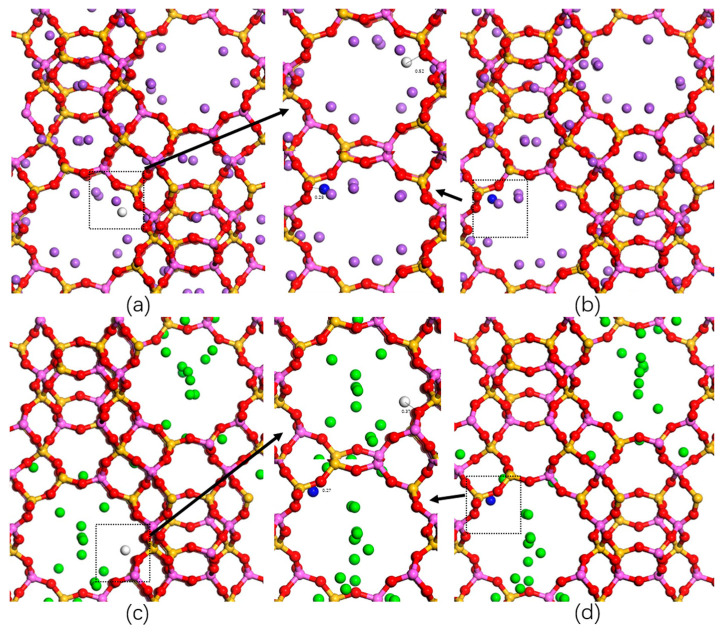
Typical binding geometry of H_2_ and N_2_ adsorbed in the super cage of the zeolite employed according to our computer simulations at 77 K. (**a**,**b**) NaX, (**c**,**d**) CaX, H_2_ molecule (white), N_2_ molecule (blue), Si-zeolite (pink), Al-zeolite (yellow), O-zeolite (red), Na^+^ cation (purple), and Ca^2+^ cation (green).

To investigate the H_2_ and N_2_ adsorption mechanism on the zeolites employed, we performed MC simulations in an NVT ensemble (with one H_2_ or N_2_ molecule per unit cell) under 1 bar at 77 K and 298 K. Figure 5 shows the interaction between H_2_ (N_2_) and the LTA-type zeolite at 77 K, and Figure 6 shows the interaction between H_2_ (N_2_) and the FAU-type zeolite at 77 K. The optimized geometry reveals a minimal O_NaA_-H distance of 0.97 Å and the minimal O_NaA_-N distance of 0.6736 Å. In Figure 6, we can observe the interaction of H_2_ (N_2_) with the FAU-type zeolite. The optimized geometry reveals a minimal O_CaX_-N distance of 0.37 Å and a minimal O_NaX_-N distance of 0.21 Å. In the preferred sites, the behavior of H_2_ is similar to that of N_2_. With similar adsorption sites, N_2_ and zeolites are closer to each other, resulting in better adsorption. The binding interaction between H_2_ and N_2_ is observed to be stronger in the FAU-type zeolite compared to the LTA-type zeolite. This observation holds true at 298 K as well, as shown in Figures S2 and S3. The adsorption site is consistent with the adsorption density pattern (Figures S4–S7). From the adsorption density figures, it is evident that H_2_ exhibits stronger adsorption density at 77 K in the vicinity of oxygen atoms in the zeolite. The adsorbate molecules interact more strongly with zeolites, and as the temperature increases, the H_2_ molecules disperse to binding sites with lower binding energies, resulting in weaker interactions with the zeolite. On the other hand, N_2_ adsorption behaves in the opposite manner, exhibiting stronger adsorption densities with the zeolite at 298 K. Significant density changes were observed in the CaA and NaX zeolites.

**Figure 7:**
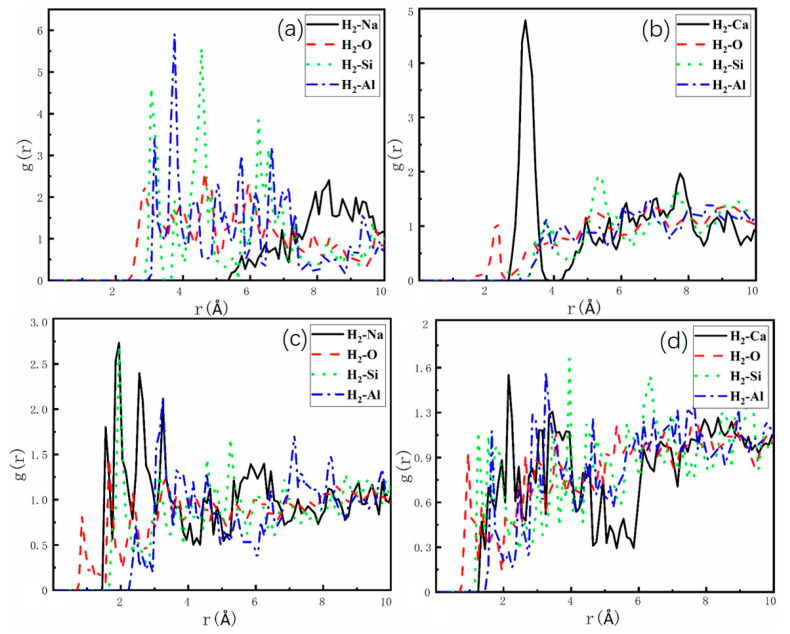
Representative interatomic guest–pore radial distribution functions (RDFs) corresponding to H_2_ at 77 K in (**a**) NaA, (**b**) CaA, (**c**) NaX, and (**d**) CaX. H_2_ molecules with Al-zeolite (blue), with Si-zeolite (green), with O-zeolite (red), and with Na^+^/Ca^2+^ cations (black).

**Figure 8:**
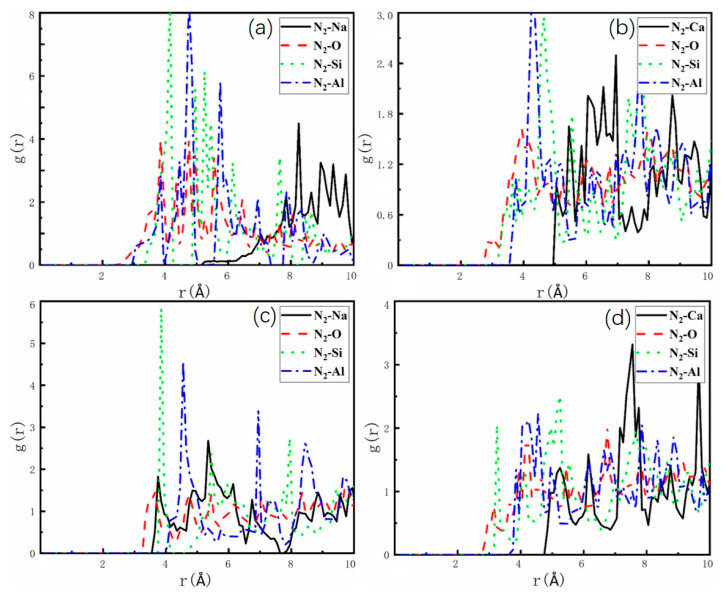
Representative interatomic guest–pore radial distribution functions (RDFs) corresponding to N_2_ at 77 K in (**a**) NaA, (**b**) CaA, (**c**) NaX, and (**d**) CaX. N_2_ molecules with Al-zeolite (blue), with Si-zeolite (green), with O-zeolite (red), and with Na^+^/Ca^2+^ cations (black).

To investigate the interaction of H_2_ and N_2_ with zeolite atoms, we employed AIMD to examine the behavior of the H_2_-N_2_ mixture (H_2_/N_2_, 50/50, *v*/*v*) within the zeolite under the NVT ensemble. The radial distribution function (RDF) was obtained by calculating the molecular trajectories of H_2_ and N_2_. Figure 7 and Figure 8 present the zonal and average densities of H_2_ and N_2_ based on O, Si, Al, and non-framework cations in LTA- and FAU-type zeolites at 1 bar and 77 K. The shape and intensity of the peaks provide evidence of the interaction sites and adsorption capacities of H_2_ and N_2_ on the zeolites. It is evident that the zeolite exhibits a stronger interaction preference for N_2_, as indicated by the local of the primary peak. In Figure 7, the initial peak occurs at 0.75 Å, indicating the highest likelihood of H_2_ being present at 0.75 Å. Similarly, in Figure 8, the radial distribution function of N_2_ displays the highest peak at 0.5 Å, indicating the highest probability of N_2_ being present at 0.5 Å. These findings clearly show that the main peaks representing the interactions between N_2_ and zeolites are higher than those of H_2_. The intensity of the main peak for H_2_ is 6, while that for N_2_ is 8, which aligns with the observation in Figure 4 regarding the larger adsorption energy for the interaction of N_2_ and zeolites. The initial peaks observed in Figure 7 and Figure 8, respectively, correspond to the oxygen atoms of framework. This suggests that the oxygen atoms of the framework assume the closest positions to the unrestricted movement of H_2_ and N_2_. Consequently, this finding supports the notion that the interaction energy between the H_2_ (N_2_) and the oxygen atoms of the framework accounts for a significant portion of the adsorption energy between the host and guest. The same principle applies at 298 K; please refer to Figures S8 and S9 for supporting evidence.

Because the dynamic diameters of H_2_ and N_2_ are similar to the effective pore diameter of the LTA- and FAU-type zeolites, diffusion plays a key role in the absorption and separation processes of H_2_ or N_2_. The diffusion of the adsorbate molecules in the zeolite is influenced not only by their dynamic diameter but also by their interaction with the oxygen atoms of the framework.

**Figure 9:**
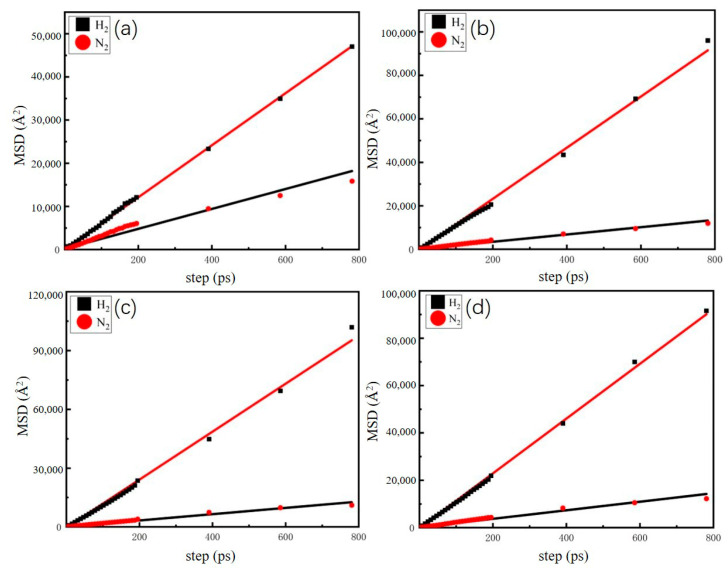
Mean square displacements for H_2_-N_2_ mixture (H_2_/N_2_, 50/50, *v*/*v*) in (**a**) NaA, (**b**) CaA, (**c**) NaX, and (**d**) CaX at 77 K. The solid line is a fitted line.

**Figure 10:**
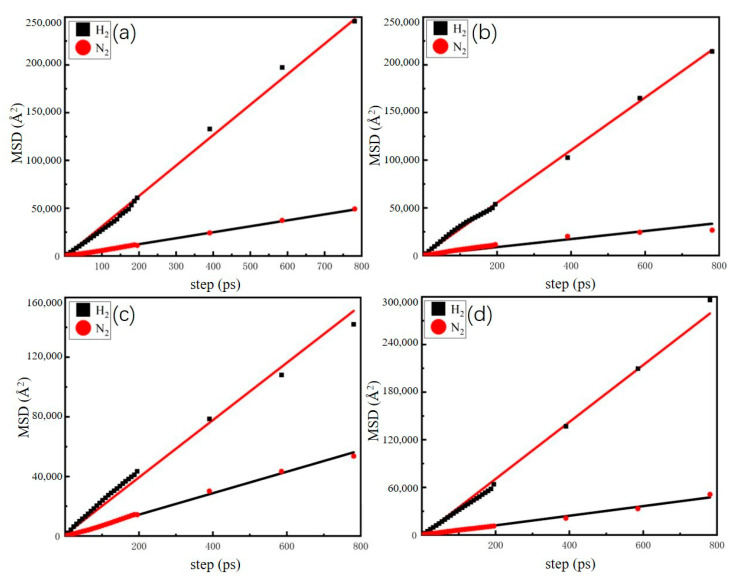
Mean square displacements for H_2_-N_2_ mixture (H_2_/N_2_, 50/50, *v*/*v*) in (**a**) NaA, (**b**) CaA, (**c**) NaX, and (**d**) CaX at 298 K. The solid line is a fitted line.

We analyzed the dynamic behavior of a H_2_-N_2_ mixture (H_2_/N_2_, 50/50, *v*/*v*) in FAU- and LTA-type zeolites at different temperatures by calculating the mean square displacements (MSDs) and the self-diffusion coefficients. Figure 9 and Figure 10 depict the relationship between the adsorbate molecular motion and equilibrium time. It is observed that the diffusion of H_2_ is greater in FAU-type zeolite than in LTA-type zeolite at 298 K. Additionally, for both FAU- and LTA-type zeolites at 298 K, the H_2_ and N_2_ diffusion capacities of Ca-FAU (Ca-LTA) are greater than those of Na-FAU (Na-LTA). During the free movement of adsorbate molecules, H_2_ diffuses more easily than N_2_ in the pores of LTA- and FAU-type zeolites. Karger conducted a diffusion study on H_2_ in NaX and NaA zeolites and found that the type of zeolite influences the diffusion process of H_2_, with the diffusion rate decreasing as the size of the zeolite pores decreases [35,36]. The linear nature of the MSD indicates the reliability of the simulation process. The self-diffusion coefficients of H_2_ and N_2_ are calculated using the Einstein relation, as shown in Equation (2):

(2) $$D=\frac{1}{6}\underset{n\to \infty }{\lim}\frac{d}{dt}\left\{\frac{1}{N_{i}}\sum_{i=1}^{N_{i}}\langle {\left|r_{i}\left(t\right)-r_{i}\left(0\right)\right|}^{2}\rangle \right\}$$

where the average is taken over time *t* for the mean square displacement of the center of mass position vectors *r* of all the molecules *N* in the system; and ‹› indicates the overall average.

The calculated results are shown in Table 3 and Table 4. Among the four types of zeolites, NaX zeolites exhibit the highest self-diffusion coefficients for H_2_ and N_2_ at 77 K, with a ratio of 7.6 (H_2_/N_2_). Similarly, CaX zeolites show the largest self-diffusion coefficients for H_2_ and N_2_ at 298 K, with a ratio of 6.54 (H_2_/N_2_). The self-diffusion coefficients of H_2_ and N_2_ in Na-FAU (Na-LTA) are lower than those in Ca-FAU (Ca-LTA).

### 2.4. Investigation of Charge Density

**Figure 11:**
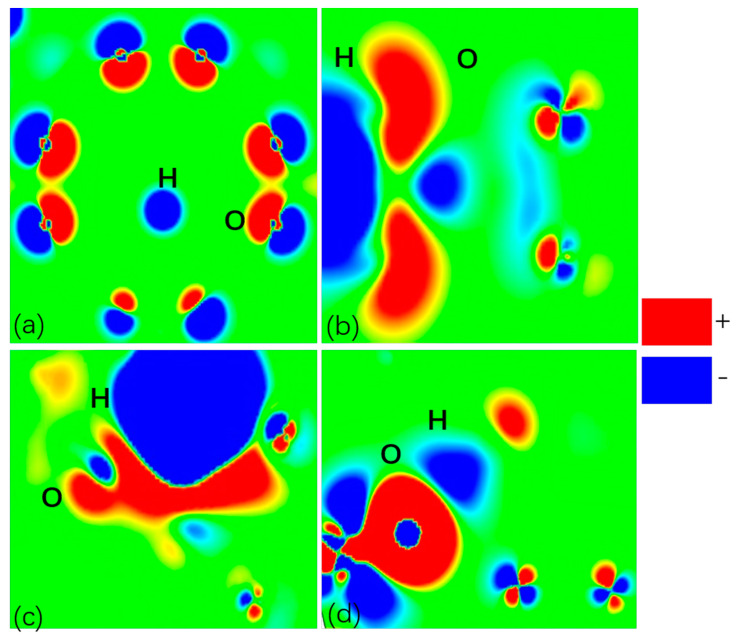
A slice of the redistribution of charge density in (**a**) NaA, (**b**) NaX, (**c**) CaA, and (**d**) CaX after adsorbing H_2_ molecules.

**Figure 12:**
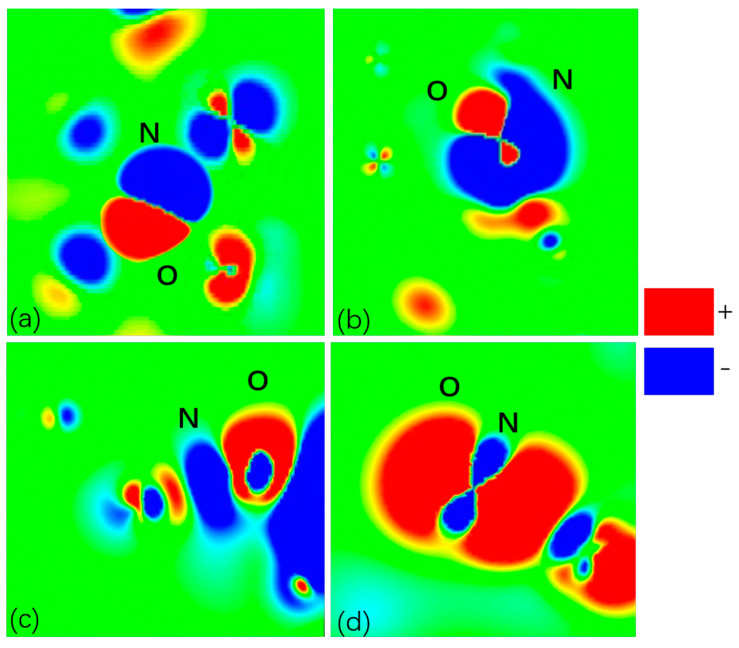
A slice of the redistribution of charge density in (**a**) NaA, (**b**) NaX, (**c**) CaA, and (**d**) CaX after adsorbing N_2_ molecules.

To investigate the effect of H_2_ and N_2_ on the chemical environment within the zeolite pores during adsorption at the atomic level, DFT calculations were performed to explore the redistribution of charge density in this system after adsorbing H_2_ or N_2_ molecules. The charge density analysis of LTA- and FAU-type zeolites is shown in Figures S10 and S11. It is clear from the figures that before the adsorption of gases, the electron cloud is concentrated around the zeolite pores. After the adsorbate molecules are adsorbed onto the zeolites, the electron density is transferred to the adsorbate molecules. The adsorption properties of the adsorbate molecules within the framework are determined using the oxygen atoms of the framework and the non-framework cations. As shown in Figure S10, due to the strong electron withdrawing ability of the zeolite atoms, the electrons of the H atoms in H_2_ molecules migrate to the framework atoms, especially the oxygen atoms. Similarly, in Figure S11, the N atoms in N_2_ donate some charges, leading to the interactions with the zeolite atoms. In comparing Figure 11 and Figure 12, it can be observed that the charge density transfers between oxygen atoms, and N_2_ is more pronounced, which is likely due to the denser electron cloud distribution of N_2_ molecules. The adsorbate molecules show an increased charge density on one side of the oxygen atoms of the zeolite framework and a decreased charge density on the other side in Figure 11 and Figure 12. H_2_ and N_2_ exhibit a blue area near the side of the oxygen atoms of the framework, indicating a decrease in charge density, while the framework atoms show a red area, indicating an increase in charge density. The electrostatic potentials of N_2_ and H_2_ in Ca-FAU (Ca-LTA) demonstrate larger values than those in Na-FAU (Na-LTA). A comparison of the analyses in Figure 11 and Figure 12 reveals that the charge transfer in the pores of LTA- and FAU-type zeolites after the adsorption of N_2_ is more pronounced than that of H_2_ and that N_2_ is more affected by the chemical environment in the zeolite pores.

## 3. Methods

### 3.1. Framework and Adsorbate: Models and Force Fields

The zeolite flexible framework has little impact on the simulation of small-molecule adsorption. In this study, the rigid structure model was employed for simulation [26]. The atoms were considered “frozen”, neglecting interatomic interactions between framework atoms to significantly shorten the simulation time [37]. The zeolite structures used in this study were taken from crystallographic files (CIFs) of the IZA database, with additional framework cations inserted for structural optimization [38]. The geometric structure was optimized using Density Functional Theory (DFT). The overall unit cell maintains a rigid structure. We analyzed the geometric structure with the Perdew–Burke–Ernzerhof (PBE) generalized gradient approximation functional, associated with the third-generation dispersion correction (DFT-D3) to avoid weak interaction, implemented in the CP2K package (8.2.0) [39,40,41]. The density cut-off was set to 500 Ry, and the overall energy converged to within 10^−6^ eV. The physicochemistry characteristics of the investigated LTA and FAU samples in this study are shown in Table 5.

The accuracy of the force field is essential for GCMC simulations. In order to validate the accuracy of our GCMC simulations, we compared the specific force field parameters of Vujić [42], General [43], Evagelia [32], Kowsari [44,45], Pantatosaki [46], and Huang [47]. The force field previously described by J. Marcos takes into consideration the influence of the dipole moment of the H_2_ molecule and the polarization of the electric field generated by the local structure of the zeolite. For hydrogen, we used a one-site point charge model, while for N_2_, we used the parameters reported by Xuan [48]. There are strong Coulomb interactions between adsorbates and non-framework cations as well as mobility of these cations. So, the location and density of the non-framework cations influence the adsorption behavior of H_2_ and N_2_ in zeolite structures [49]. The cation Ca^2+^ parameters reported by Du and the cation Na^+^ parameters reported by Farida were employed to describe interactions in this work [45,50]. The force field parameters and partial charges are presented in Table 6.

The simulation accuracy of classical force fields is not sufficient when the polarization effect of N_2_ is neglected. To enhance the simulation accuracy, the polarization force field is introduced by scaling the Lennard-Jones (L-J) interaction between N_2_ and zeolite atoms with a parameter λ ∈ [0, 1] [51,52]. The atomic polarizabilities used in this study were obtained from the literature using Equation (3). For this study, *λ* was set to 0.4 by fitting the experimental data to the simulation results.

(3) $$\varepsilon_{i}^{scaled}=\varepsilon_{i}\cdot \frac{\left(1+\lambda \right)-\frac{\alpha_{i}}{\alpha_{max}}}{\left(1+\lambda \right)-\frac{\alpha_{i}}{\alpha_{max}}\cdot \lambda }$$

where *λ* is a scaling factor between 0 and 1 used for rescaling the Lennard-Jones energy parameters; *α_i_* means the polarizability of atom_i_; *α_max_* means the max polarizability; and *ε_i_* means the initial force field parameter. *λ* = 1 means that N_2_ has the same interaction potential energy function with other molecules, and *λ* = 0 means that N_2_ does not interact with other molecules.

### 3.2. Simulation Details

The separation of H_2_ and N_2_ adsorption in LTA- and FAU-type zeolites was performed using the RASPA code [53,54]. The cut-off radius was set to half the length of the cell edge in the simulation box, without considering tail corrections, and by applying periodic boundary conditions to maintain a three-dimensional spatial structure. Only cations and adsorbate molecules were allowed to move, while the zeolite structure remained rigid. In these simulations, the following trial moves were used for the cations: translation (50%) and random (50%). For the H_2_ and N_2_ molecules, the trial moves were carried out: translation (20%), rotation (20%), insertion (20%), and random (40%). The total number of cations remained constant during the simulations, and only translation movements and regrow were considered for this type of particle. Based on the reported crystallographic locations of Na^+^, Na^+^ molecules were expected to enter the sodality cages in FAU-type zeolites, but not in LTA-type zeolites [55]. Therefore, blocking spheres were exclusively used for LTA-type zeolites in our investigation, with the ionic radius of Na^+^ (r_Na_^+^ = 1.16) serving as the probe size. The simulations were performed for 5 × 10^5^ cycles, with 25,000 initialization cycles and 20,000 equilibration cycles. The final simulation result was obtained by averaging the results of the two simulation processes. The excess adsorption data were used for investigation [56]. The excess adsorption capacity (*n_exc_*) was obtained by relating the absolute adsorption capacity (*n_abs_*) to the pore volume of the adsorbent (*V_g_*) and the molar density of the native gas phase (*ρ_g_*), using Equation (4):

(4) $$n_{exc}=n_{abs}-V^{g}\rho^{g}$$

In the He-void (ϕHe) calculations, the particle method with the Rosenbluth algorithm was used [57]. The largest cavity diameter (LCD), pore volume, and accessible surface area (ASA) were determined using Zeo^++^ software packages (0.3) [58]. The interaction between the adsorbates and the zeolite atoms was calculated based on electrostatic and van der Waals force interactions. The electrostatic interactions were computed using the Coulomb force, while the interatomic interaction parameters were determined using Lorentz–Berthelot mixing rules [59]; Equation (5) was used for this calculation:

(5) $$\varepsilon_{ij}=\sqrt{\varepsilon_{i}\varepsilon_{j}},\;\sigma_{ij}=\frac{\sigma_{i}+\sigma_{j}}{2}$$

where *σ_ij_* and *ε_ij_* are the L-J potential parameters.

Van der Waals interactions were described using the 12-6 Lennard-Jones (L-J), and the Ewald method was employed to calculate long-range electrostatic interactions with a relative accuracy of 10^−6^ [60]; Equation (6) was used for this calculation:

(6) $$E_{LJ}\left(r_{ij}\right)=4\varepsilon_{ij}\left[{\left(\frac{\sigma_{ij}}{r_{ij}}\right)}^{12}+{\left(\frac{\sigma_{ij}}{r_{ij}}\right)}^{6}\right]$$

where *r_ij_* is the interatomic distance between the i and j atoms.

An MD simulation was performed using the RASPA code. Molecular dynamic runs of H_2_-N_2_ (H_2_/N_2_, 50/50, *v*/*v*) molecules were conducted in a 25 × 25 × 25 Å box at 77 and 298 K, with periodic boundary conditions imposed in all directions. The MD simulation was run for 1 ns with a time step of 0.5 fs in the NVT ensemble. The simulations included 2 × 10^6^ cycles, with 2000 initialization cycles and 20,000 equilibration cycles.

To explore the distribution of H_2_ and N_2_ molecules in zeolites, the ab initio molecular dynamic (AIMD) simulation was employed using the CP2K program (8.2.0). The simulations employed the Gaussian plane wave method, implemented in the Quickstep module [61], with the input file using the Multiwfn code [62]. The simulation was performed in the NVT ensemble using the BASIS_MOLOPT basis set [63] and GTH_POTENTIALS pseudopotential [64]. The employed density functional was the PBE with the DFT-D3 dispersion corrections. The CSVR thermostat with a temperature relaxation time of 0.5 ps was used to keep the temperature at 298 K during the simulations [65]. All reported properties were averaged over at least 500 ps, and the time step was set to 0.5 fs.

## 4. Conclusions

This study investigated the adsorption of H_2_ and N_2_ single components in Na^+^/Ca^2+^ ion-exchanged LTA- and FAU-type zeolites using GCMC and MD simulations. The adsorption isotherms obtained by the simulations correspond well with the experimental results. It is observed that the Ca^2+^ zeolites display a higher H_2_ and N_2_ adsorption capacity. The IAST predicts the adsorption selectivity of H_2_-N_2_ mixture in LTA- and FAU-type zeolites. The findings indicate that the selectivity of N_2_/H_2_ is greater for NaX at 77 K and NaA at 298 K. Additionally, the kinetic simulation of H_2_-N_2_ mixture diffusion demonstrated that H_2_ exhibits the fastest diffusion rate in NaX.

This study on adsorption sites for H_2_ and N_2_ on LTA- and FAU-type zeolites shows that major adsorption sites are located near framework oxygen atoms and non-framework cations. For the non-framework cations, Na^+^/Ca^2+^, the findings regarding adsorption energy and radial distribution function suggest that the Ca^2+^ zeolites exhibit a stronger interaction energy with H_2_ and N_2_. Moreover, there is an apparent interaction between the zeolite and N_2_ from the redistribution of charge density. This results in a relatively large charge transfer, leading to a stronger adsorption capacity.

## Figures and Tables

**Table 1:** The physical parameters of H_2_ and N_2_.

Molecular	Kinetic Diameter (Å)	Polarizability (Å)	Dipole Moment (D)	Quadruple Moment (D Å)
H_2_	2.89	0.8	0	0
N_2_	3.6	1.74	0	1.18

**Table 2:** Heats of adsorption at infinite dilution of H_2_ and N_2_ on zeolites.

Molecular	Zeolite	Experiment Adsorption Heat (kJ/mol)		Simulation Adsorption Heat (kJ/mol)	
		77 K	298 K	77 K	298 K
H_2_	NaA	−10.59	−4.47	−9.23	−4.50
	CaA	−7.40	−4.32	−8.12	−5.51
	NaX	−10.54	−4.30	−9.47	−4.94
	CaX	−6.39	−4.51	−5.25	−5.99
N_2_	NaA	−17.07	−16.71	−17.16	−18.65
	CaA	−18.06	−15.01	−21.87	−17.23
	NaX	−18.98	−14.19	−17.29	−15.07
	CaX	−11.73	−11.05	−15.51	−11.69

**Table 3:** Computed self-diffusion coefficients of H_2_-N_2_ mixture (H_2_/N_2_, 50/50, *v*/*v*) in LTA- and FAU-type zeolites at 77 K.

Zeolites	NaA	CaA	NaX	CaX
H_2_ (Å^2^/ps)	10.1	19.6	20.3	19.3
N_2_ (Å^2^/ps)	3.8	2.8	2.7	3.0
H_2_/N_2_	2.6	7.0	7.6	6.4

**Table 4:** Computed self-diffusion coefficients of H_2_-N_2_ mixture (H_2_/N_2_, 50/50, *v*/*v*) in LTA- and FAU-type zeolites at 298 K.

Zeolites	NaA	CaA	NaX	CaX
H_2_ (Å^2^/ps)	52.8	46.0	32.2	57.9
N_2_ (Å^2^/ps)	10.4	7.0	12.2	10.1
H_2_/N_2_	5.1	6.5	2.7	5.7

**Table 5:** Physicochemistry characteristics of LTA and FAU Systems.

	NaA	CaA	NaX	CaX
Accessible pore volume (cm^3^/g)	0.28	0.3	0.261	0.31
Supercage pore size (Å)	4	5	7.4	8
Cation	Na	Ca	Na	Ca
Number of cations (N)	96	48	88	44
Si/Al	1	1	1	1

**Table 6:** Lennard-Jones interactions and partial charges.

Atom Types	ε (K)	σ (Å)	Q (e)	CITE
H_2_	38	2.92	-	[48]
N_2_	95.2	3.75	-	[49]
H_2_-Si	39	2.8	2.234	[48]
H_2_-Al	42.5	2.95	2.089	[48]
H_2_-O	47	3.08	−1.307	[48]
Na	8	3.5	0.8	[50]
Ca	78	2.98	2	[45]

## Data Availability

The data presented in this study are available upon request from the corresponding author.

## Supplementary Materials

The following supporting information can be downloaded at https://www.mdpi.com/article/10.3390/molecules29153686/s1: Table S1: The deviation between the molecular simulations and the experiments for H_2_ and N_2_ on LTA- and FAU-type zeolites; Table S2: Experimental consistency of adsorption isotherm from Langmuir isotherm for H_2_ and N_2_ on LTA- and FAU-type zeolites; Figure S1: Comparison between N_2_/H_2_ selectivity and IAST; Figure S2: Typical binding geometry of H_2_ and N_2_ adsorbed in super cage of zeolite employed according to our computer simulations at 298 K; Figure S3: Typical binding geometry of H_2_ and N_2_ adsorbed in super cage of zeolite employed according to our computer simulations at 298 K; Figures S4 and S5: Adsorption density of H_2_; Figures S6 and S7: Adsorption density of H_2_; Figure S8: Representative interatomic guest-pore radial distribution functions (RDFs) corresponding to H_2_ at 298 K; Figure S9: Representative interatomic guest–pore radial distribution functions (RDFs) corresponding to N_2_ at 298 K; Figures S10 and S11: Redistribution of charge density.
