# Supplementary material for: Competitive Adsorptive Mechanism of H2/N2 in LTA/FAU Zeolites by Molecular Simulations and Experiments
Source: Molecules. 2024 Aug 3;29(15):3686. doi: 10.3390/molecules29153686 (PMC11314403; doi:10.3390/molecules29153686)
Supplement: Supplementary file 1 [file molecules-29-03686-s001.zip › molecules-3048511-supplementary.pdf]

Supporting Information

Competitive Adsorptive Mechanism of H_2/N_2 in LTA/FAU Zeolites by Molecular Simulations and Experiments

Zixu Dong, Zhilu Wang, Lina Zhang, Qiang Fu * and Ming Wang *

School of Chemistry and Chemical Engineering, Shandong University of Technology, Zibo 255049, China; 21406020666@stumail.sdut.edu.cn (Z.D.); sevenwangzl@163.com (Z.W.); zln20000521@163.com (L.Z.)

* Correspondence: fuqiang@tju.edu.cn (Q.F.); wangmingmw@sdut.edu.cn (M.W.)

Table S1. The deviation between the molecular simulations and the experiments for H_2 and N_2 on LTA and FAU type zeolites.

	RAD (H_2 loading)		RAD (N_2 loading)	
	77 K	298 K	77 K	298 K
NaA	0.1432	0.2395	0.1070	0.2102
CaA	0.0508	0.1136	0.0206	0.2671
NaX	0.1718	0.1869	0.0280	0.1786
CaX	0.1821	0.0826	0.0450	0.1380

Table S2. The experimental consistency of adsorption isotherm from Langmuir isotherm for H_2 and N_2 on LTA and FAU type zeolites.

	**Zeolite-H_2**						**Zeolite-N_2**					
	**77 K**			**298 K**			**77 K**			**298 K**		
	q_m	b_i	R^2	q_m	b_i	R^2	q_m	b_i	R^2	q_m	b_i	R^2
LTA-Na	5.7	3.7	0.98203	0.6	0.2	0.97205	15.3	1	0.99390	2.1	0.57	0.98966
LTA-Ca	8.5	4.0	0.99405	2.2	0.86	0.98897	7.7	1.5	0.98664	4.8	0.47	0.97149
FAU-Na	4.2	1.3	0.99378	0.6	0.47	0.99910	8.2	3.4	0.94025	4.5	0.66	0.97707
FAU-Ca	5.1	1.4	0.99614	4.1	0.5	0.99504	5.8	2.2	0.97148	2.7	0.22	0.99967

The Langmuir model is presented in Equation（1）:

$$q_i^* = \frac{q_{m,i} b_i P}{1+b_i P} \qquad (1)$$

Where b_i is adsorption affinity, bar^{-1}. q_i^* is adsorbed phase concentration in equilibrium with bulk gas of component i, $mol{\cdot}kg^{-1}$. $q_{m,i}$ is specific saturation adsorption capacity of component i, $mol{\cdot}kg^{-1}$. P is pressure, *bar.*

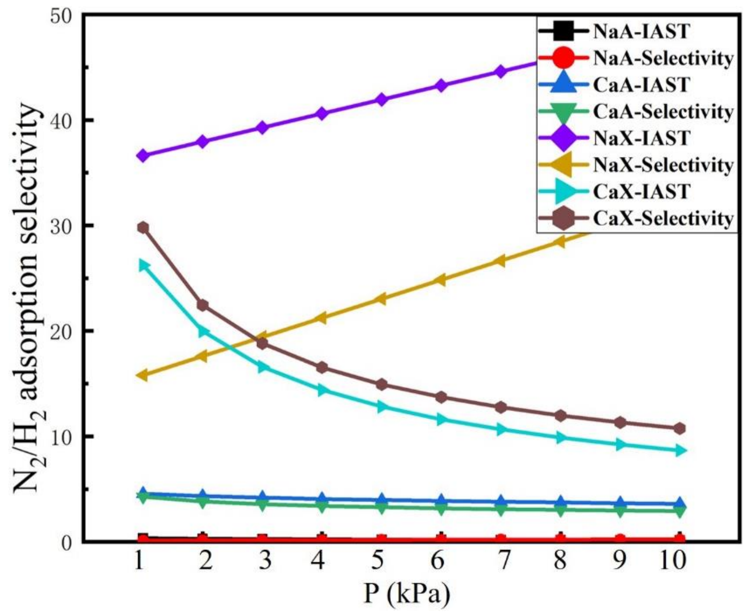

Figure S1. The N_2/H_2 selectivity obtained from binary N_2-H_2 mixture by GCMC simulations and IAST.

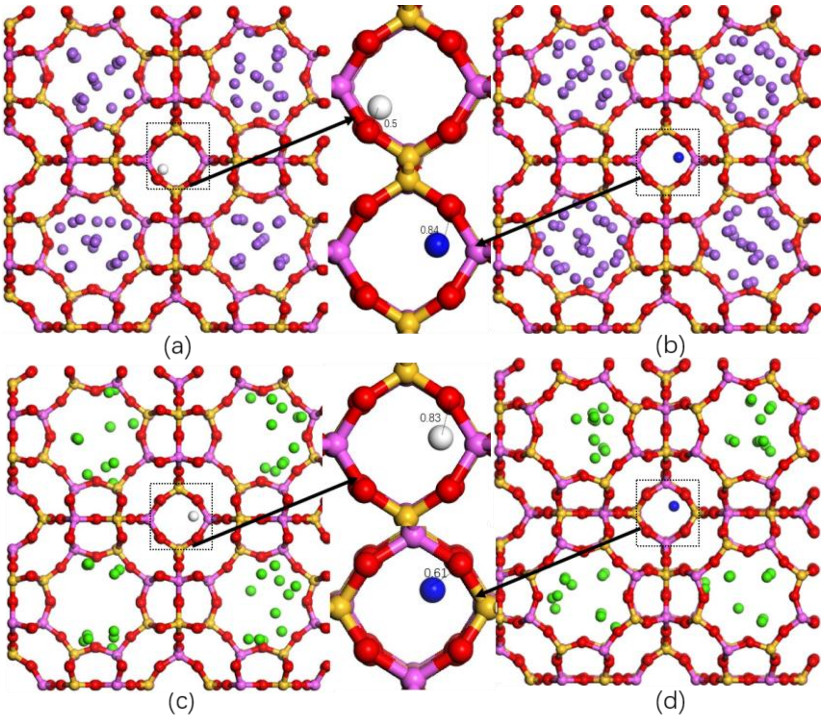

Figure S2. Typical binding geometry of H_2 and N_2 adsorbed in the super cage of zeolite employed according to our computer simulations at 298 K. **(a)(b)** NaA, **(c)(d)** CaA, H_2 molecule (white), N_2 molecule (blue), Si-zeolite (pink), Al-zeolite (yellow), O-zeolite (red), Na^+ cation (purple), Ca^{2+} cation (green).

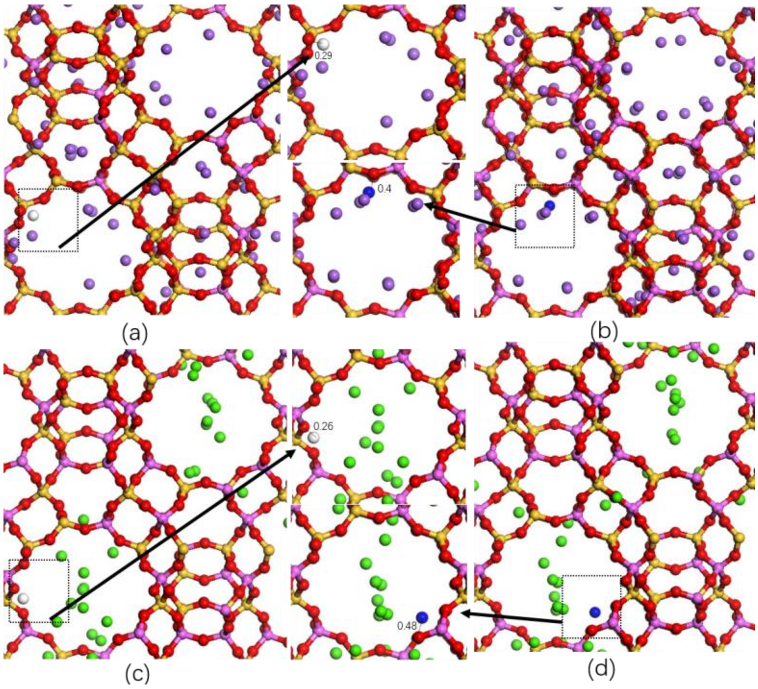

Figure S3. Typical binding geometry of H_2 and N_2 adsorbed in the super cage of zeolite employed according to our computer simulations at 298 K. **(a)(b)** NaX, **(c)(d)** CaX, H_2 molecule (white), N_2 molecule (blue), Si-zeolite (pink), Al-zeolite (yellow), O-zeolite (red), Na^+ cation (purple), Ca^{2+} cation (green).

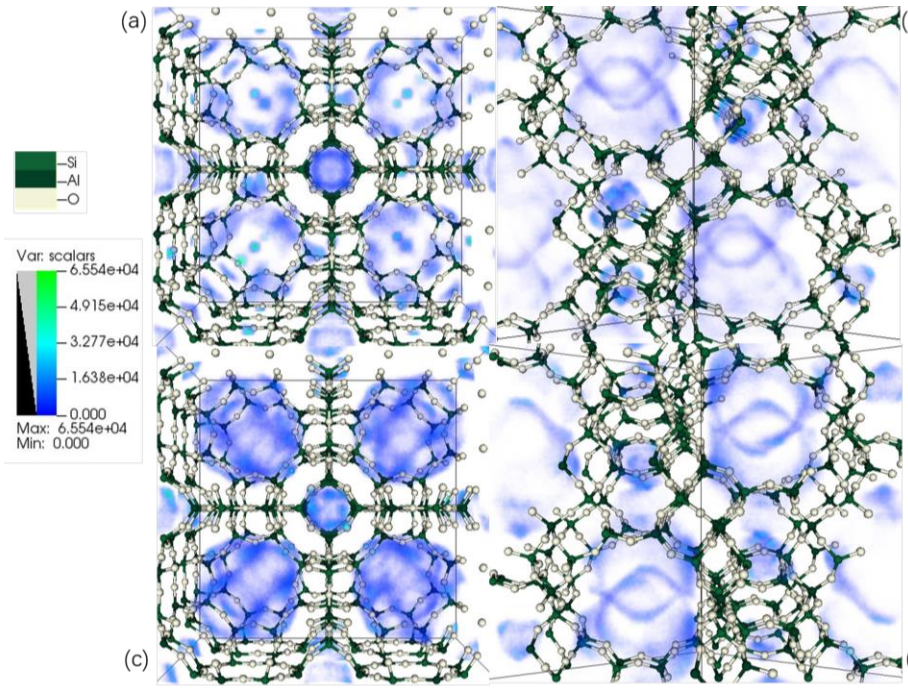

Figure S4. Adsorption density of H_2 at 77 K,1bar in **(a)** NaA, **(b)** NaX, **(c)** CaA, **(d)** CaX.

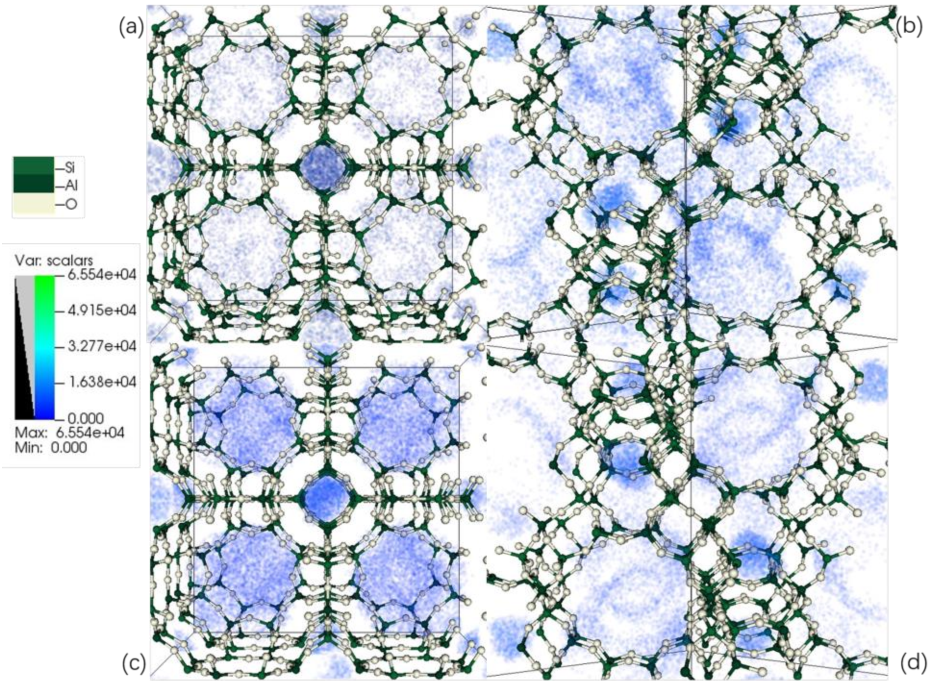

Figure S5. Adsorption density of H_2 at 298 K,1bar in **(a)** NaA, **(b)** NaX, **(c)** CaA, **(d)** CaX.

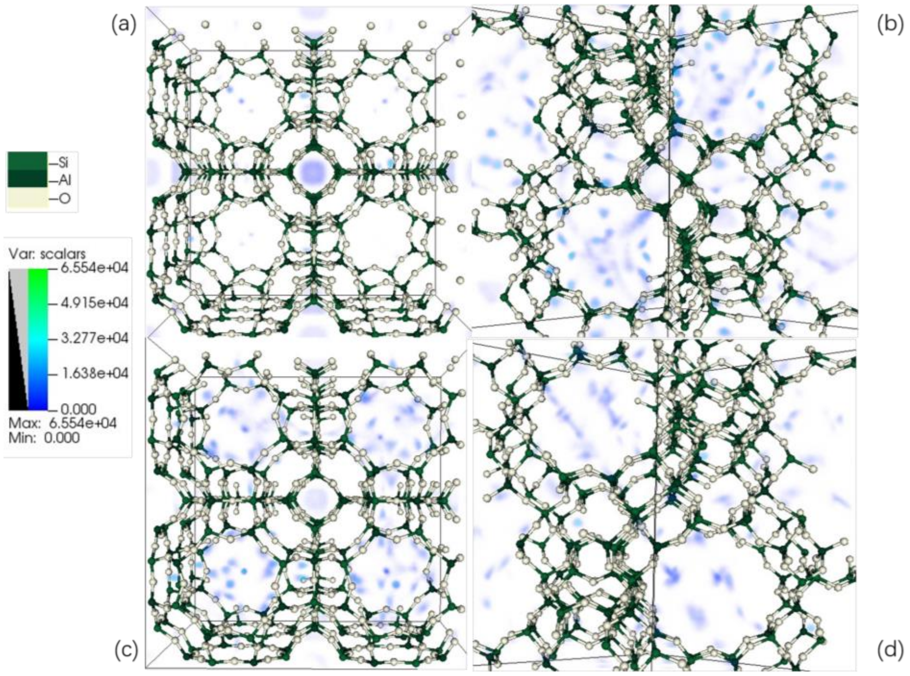

Figure S6. Adsorption density of N_2 at 77 K,1bar in **(a)** NaA, **(b)** NaX, **(c)** CaA, **(d)** CaX.

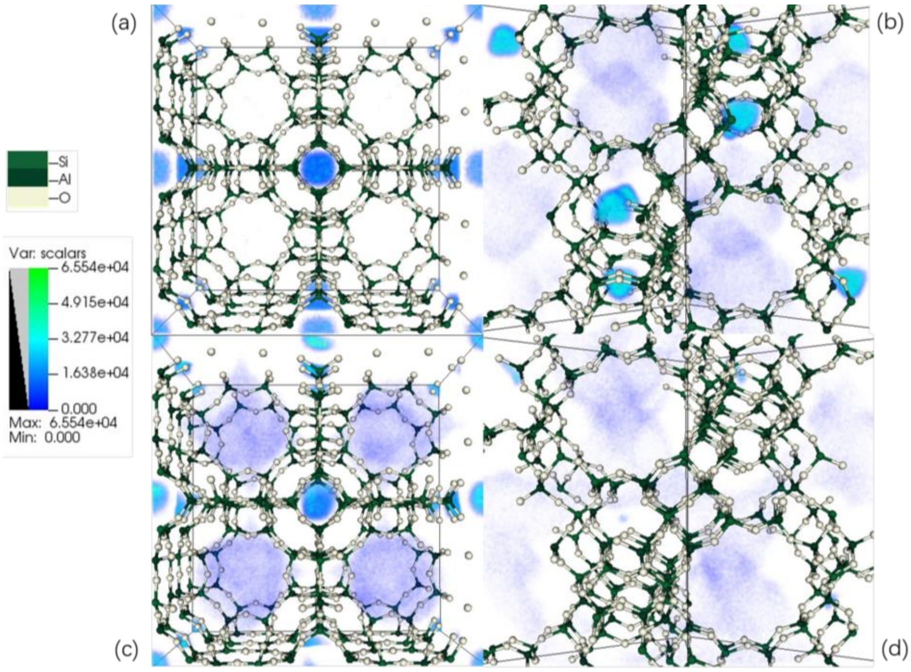

Figure S7. Adsorption density of N_2 at 298 K,1bar in **(a)** NaA, **(b)** NaX, **(c)** CaA, **(d)** CaX.

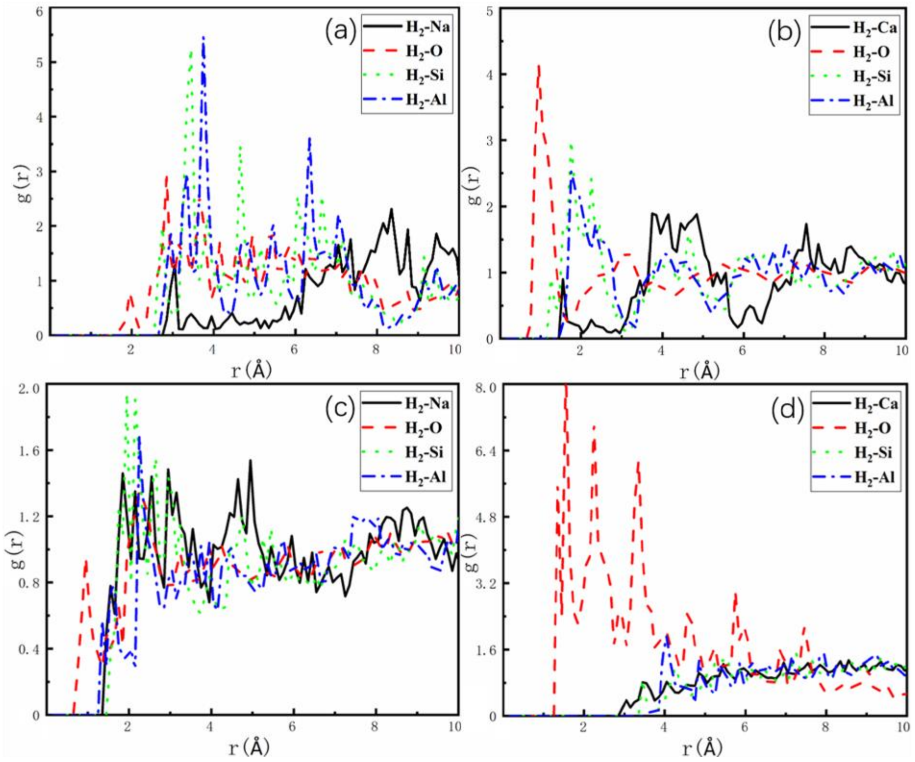

Figure S8. Representative interatomic guest-pore radial distribution functions (RDFs), correspond to H_2 at 298 K in **(a)** NaA, **(b)** CaA, **(c)** NaX, **(d)** CaX. H_2 molecule with Al-zeolite (blue), with Si-zeolite (green), with O-zeolite (red), with Na^+/Ca^{2+} cations (black).

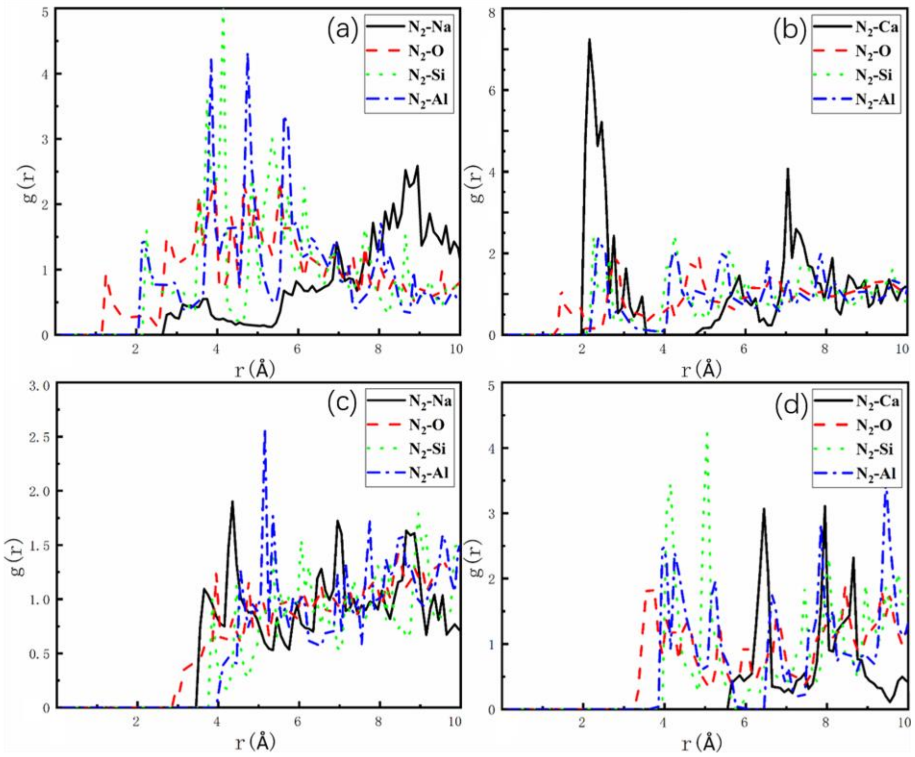

Figure S9. Representative interatomic guest-pore radial distribution functions (RDFs), correspond to N_2at 298 K in **(a)** NaA, **(b)** CaA, **(c)** NaX, **(d)** CaX. N_2 molecule with Al-zeolite (blue), with Si-zeolite (green), with O-zeolite (red), with Na^+/Ca^{2+} cations (black).

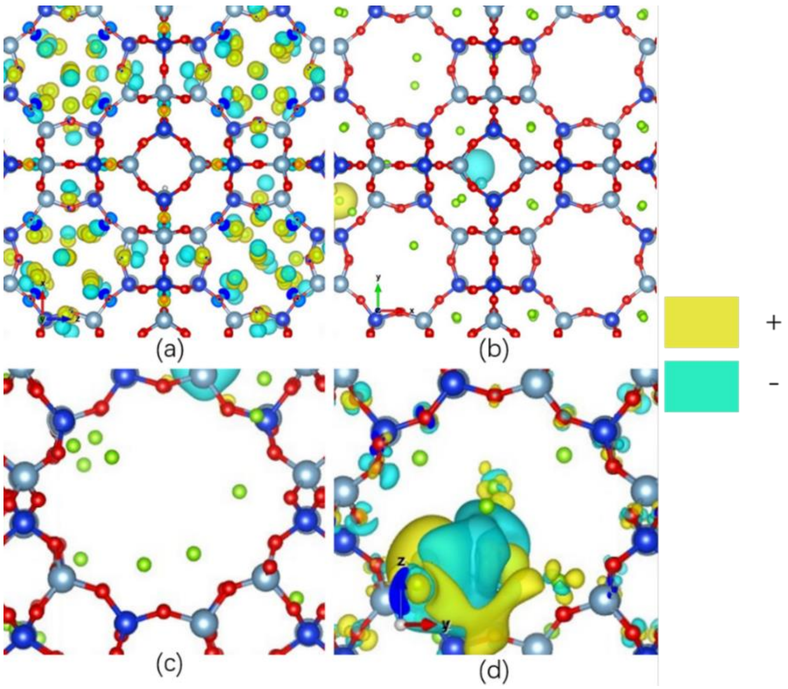

Figure S10. The redistribution of charge density in **(a)** NaA, **(b)** NaX, **(c)** CaA, **(d)** CaX after adsorbing H_2 molecules, Color code: red, O; grey, Si; blue, Al; green, cation.

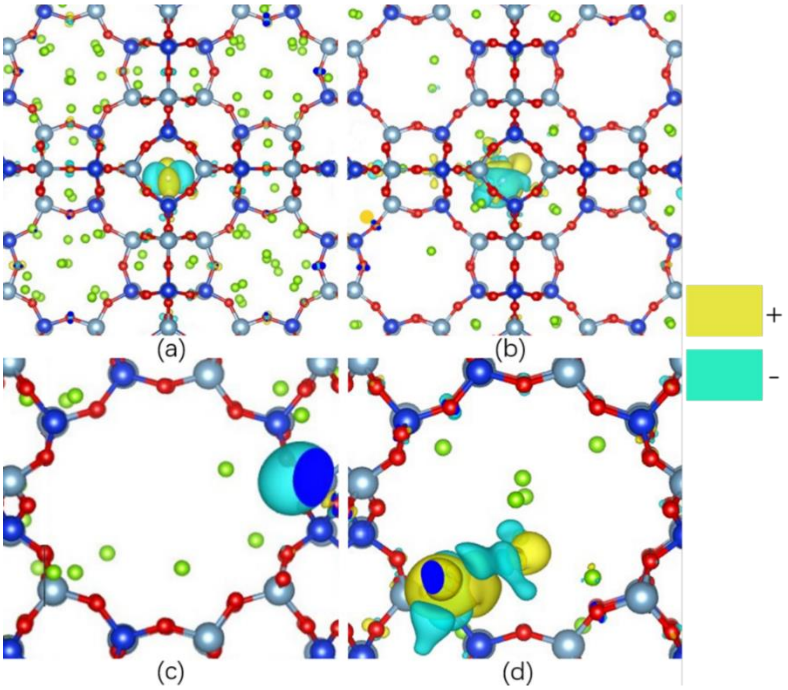

Figure S11. The redistribution of charge density in **(a)** NaA, **(b)** NaX, **(c)** CaA, **(d)** CaX after adsorbing N_2 molecules, Color code: red, O; grey, Si; blue, Al; green, cation.
